# Enterovirus D68–Associated Severe Pneumonia, China, 2014

**DOI:** 10.3201/eid2105.150036

**Published:** 2015-05

**Authors:** Tiegang Zhang, Lili Ren, Ming Luo, Aihua Li, Cheng Gong, Meng Chen, Xiali Yu, Jiang Wu, Ying Deng, Fang Huang

**Affiliations:** Beijing Center for Disease Prevention and Control, Beijing, China (T. Zhang, M. Luo, A. Li, C. Gong, M. Chen, X. Yu, J. Wu, Y. Deng, F. Huang);; Institute of Pathogen Biology, Beijing (L. Ren)

**Keywords:** enterovirus, severe pneumonia, genome, viruses, China, EV-D68, respiratory infections

**To the Editor:** Over the past 4 years, outbreaks caused by enterovirus type D68 (EV-D68) infection have occurred in many parts of the world (*1*); this virus can cause severe respiratory tract infections (RTIs) in children. This public health concern has been boosted by the recent outbreaks of EV-D68 infection in the United States (http://www.cdc.gov/non-polio-enterovirus/outbreaks/EV-D68-outbreaks.html). Outbreaks associated with novel EV-D68 have also been reported during 2006–2012 in China (*2*,*3*). However, since 2012, no EV-D68 infections in China have been reported. Whether the EV-D68 outbreaks in the United States affected those in China is unclear. Continuous characterization of EV-D68 epidemics is therefore necessary for purposes of early alert and for facilitating control measure decisions.

To determine EV-D68 prevalence in China, we screened for EV-D68 infections in 2014 in Beijing, China. We tested patients with RTI during August–November 2014, reported by the Respiratory Virus Surveillance System, established by Beijing Center for Disease Prevention and Control. The System covers 30 sentinel hospitals in all 16 districts of Beijing. We obtained 1,478 clinical specimens (1,034 nasopharyngeal swab and 444 sputum). Patient ages ranged from 8 months to 93 years (median 33.5 years, mean 37.9 years). Enteroviruses and other known respiratory viruses were detected by real-time PCR (*4*). A total of 70 enterovirus-positive samples were identified. Other respiratory viruses detected were 89 rhinoviruses, 87 influenza viruses, 70 human parainfluenza viruses (types 1–4), 43 human coronaviruses, 26 respiratory syncytial viruses, 29 adenoviruses, 9 bocaviruses, and 2 metapneumoviruses. Among the EV-positive samples, 1 was positive for EV-D68 according to PCR amplification of the viral protein 1 (VP1) gene (*5*); no other respiratory viruses were detected in this patient. 

The EV-D68–positive patient was a 5-year-old girl with no underlying disease. On August 5, 2014, she had fever (highest temperature 40°C), cough, breathing difficulty, abdominal pain, diarrhea, and vomiting. Her condition deteriorated, and on August 11, she was hospitalized in Beijing Children’s Hospital with a diagnosis of severe pneumonia. A nasopharyngeal swab sample was collected at the time of admission. She was released on August 16, after receiving symptomatic supportive treatment in the general ward; she had not required mechanical ventilation. She and her family had no history of travel in the months before she became ill.

To further characterize this virus strain, Beijing-R0132, we amplified the genome sequence directly from a nasopharyngeal swab sample by using overlapping primers designed according to the reference sequence (GenBank accession no. KM892501; Technical Appendix). The termini of the genome were independently determined by using the RACE System (Invitrogen, Carlsbad, CA, USA) according to the manufacturer’s protocol. Each amplicon was sequenced 4 times by using the Sanger method, and the forward and reverse sequences agreed well. Sequences were assembled by using DNAStar software (Lasergene, Madison, WI, USA) and deposited in GenBank (accession no. KP240936). The genome of Beijing-R0132 was 7,334 nt long, including 699 nt in 5′-untranslated regions (5′-UTRs), 6,567 nt in open reading frame (ORF), and 68 nt in 3′-UTR. Beijing-R0132 shared 96% nt sequence identity with the virus circulating in the United States in 2014, US/CO/14-60. In contrast to the prototype EV-D68 (Fermon strain, AY426531), deletions of the CTCAAAACCTCCAGTACATAACA sequence in the 5′-UTR and TTATTTATAACA sequence in the front of the ORF of Beijing-R0132 were observed, corresponding with nt 682–704, and nt 721–732 of the Fermon strain, which were similar to those identified in the United States in 2014.

We then used MEGA software version 6.06 (http://www.megasoftware.net) to analyze the phylogeny of the whole genome and the VP1 gene with EV-D68 sequences available in GenBank (Figure). Beijing-R0132 was clustered with most of the EV-D68 strains that circulated throughout the United States during 2014. The strains identified from Beijing in 2008, represented by BCH895A/2008, belong to another distinct lineage according to genome phylogeny (Figure, panel A). Similar relationships were observed in the phylogenic tree of the VP1 gene (Figure, panel B). Beijing-R0132 and some EV-D68 strains from China identified in 2011 grouped with most of the strains obtained from the United States in 2014. These findings demonstrate that the EV-D68 strain circulating in Beijing was closely related to strains circulating in the United States.

**Figure:**
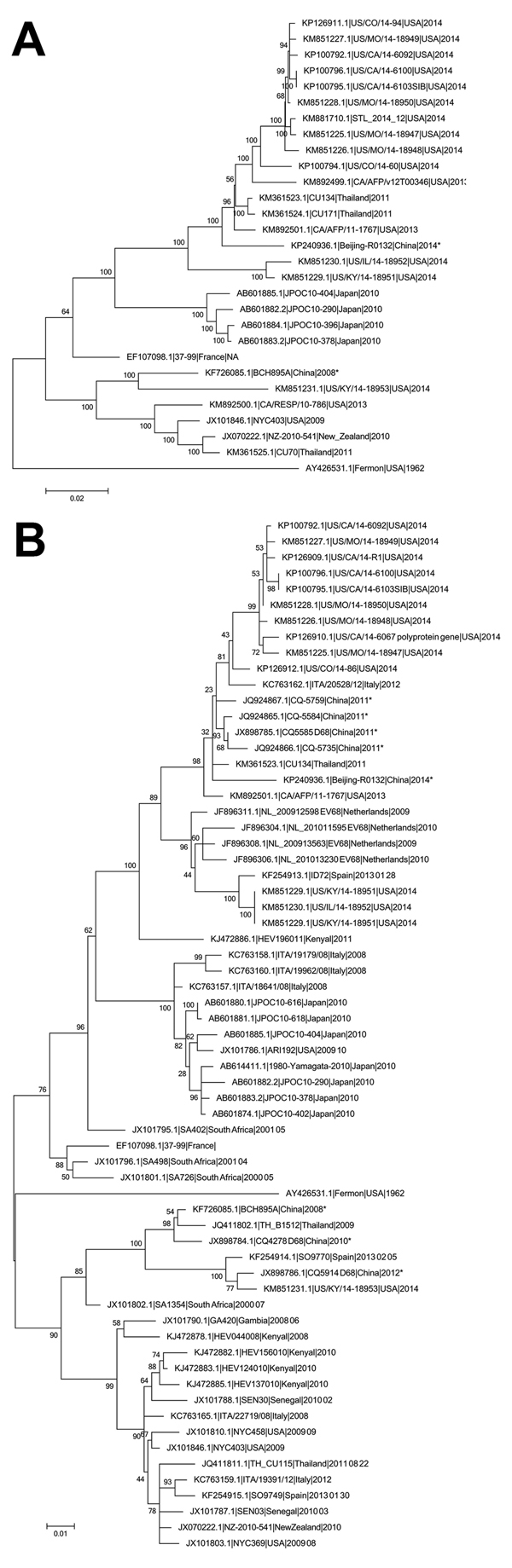
Phylogenetic analysis of enterovirus D68 (EV-D68) from different locations. The phylogenetic relationships of all complete or near-complete EV-D68 genomes (A) or representative viral protein 1 sequences from different countries (B) were estimated by using the maximum-likelihood method with 100 replicates bootstrapped by using MEGA (http://www.megasoftware.net). Bootstrap values were indicated on each tree. EV-D68 strains from China are indicated with an asterisk. GenBank accession numbers are shown for each EV-D68 strain. Scale bars indicate nucleotide substitutions per site.

The origin of the 2014 EV-D68 outbreaks in the United States is unclear. This Beijing-R0132 genome sequence provides information for tracking EV-D68 as it spreads throughout the world and for evaluating the sequence diversity in circulating EV-D68 strains. In contrast to EV-D68 detection during the outbreaks in the United States, positive detection of EV-D68 in this study was limited, although the circulating virus strains were closely related. The reason for this disparity warrants further investigation. However, the severe pneumonia caused by EV-D68 reported here underlies the need for intensive attention to surveillance and control of EV-D68 in vulnerable populations, such as young children. In addition, for a megacity with very high population density and mobility, such as Beijing, the citywide implementation of the Respiratory Virus Surveillance System is critical for monitoring the epidemic or potential outbreaks of EV-D68 and other respiratory viruses and for enabling early warning.

Technical Appendix Primers used for amplification and sequencing of the genome of Beijing-R0132.
