# Supplementary material for: Enterovirus D68–Associated Severe Pneumonia, China, 2014
Source: Emerg Infect Dis. 2015 May;21(5):916–8. doi: 10.3201/eid2105.150036 (PMC4412250; doi:10.3201/eid2105.150036)
Supplement: Technical Appendix — Primers used for amplification and sequencing of the genome of Beijing-R0132. [file 15-0036-Techapp-s1.pdf]

Article DOI: http://dx.doi.org/10.3201/eid2105.150036

Enterovirus D68–Associated Severe Pneumonia, China, 2014

Technical Appendix

Technical Appendix Table. Primers used for amplification and sequencing of the genome of enterovirus D68 strain Beijing-R0132

Primer	Sequences, 5′→3′	Position*	Amplicons
P-EV68-1-F	TTAAAACAGCCTTGGGGTTG	1–20	833 bp
P-EV68-1-R	TGGCTGAAGCCGCATAACTA	833–814	
P-EV68-2-F	AACATTGCCACAAATGGATC	758–777	917 bp
P-EV68-2-R	GTATGTTGGGACACCTTGAGTAA	1675–1653	
P-EV68-3-F	TACGCGTACAATGTCAAGTATG	1570–1591	899 bp
P-EV68-3-R	ACCACACCAAGTTCAGCGTTA	2469–2449	
P-EV68-4-F	CCTTGATAGGGTTCATAGCAGC	2292–2313	1007 bp
P-EV68-4-R	CTGGACCAGTGGTCACTA	3299–3282	
P-EV68-5-F	CACCACGAACCCTGCCATA	3159–3177	871 bp
P-EV68-5-R	CTGTCTAGGTACATAAGGAATGCC	4030–4007	
P-EV68-6-F	ACACTAGCATTGTTGGGATGC	3938–3958	817 bp
P-EV68-6-R	AATCTGCGTGACAAAGCCT	4755–4737	
P-EV68-7-F	GGAACTCTGTACACCAGTCCAT	4658–4679	893 bp
P-EV68-7-R	AGTCAAATCTCTAAGCGCACAC	5551–5530	
P-EV68-8-F	TATGATAGGGTAGCGGTCATC	5444–5464	819 bp
P-EV68-8-R	TCCCTTGTAGTAAGTAAGGGAAT	6263–6241	
P-EV68-9-F	TCAGTGTGGATCCCATACC	6159–6177	591 bp
P-EV68-9-R	GGCATCCCACCATTAACTATATAT	6750–6727	
P-EV68-10-F	ATGCTAGTTTGTCACCAGTGTG	6603–6624	740 bp
P-EV68-10-R	GCCCCCAAGTGACCAAAATT	7343–7324	
P-EV68-5-RACE-R	GTCCAGACTCATCGACC	333–317	5′-UTR†
P-EV68-3-RACE-F	GATATTTCAGAGCAGATGATC	7017–7037	3′-UTR

*Nucleotide positions indicated are those corresponding to GenBank accession no. KM892501.

†UTR, untranslated region.
